# Bi-Directional Piezoelectric Multi-Modal Energy Harvester Based on Saw-Tooth Cantilever Array

**DOI:** 10.3390/s22082880

**Published:** 2022-04-08

**Authors:** Andrius Čeponis, Dalius Mažeika, Artūras Kilikevičius

**Affiliations:** 1 Department of Engineering Graphics, Faculty of Fundamental Sciences, Vilnius Gediminas Technical University, Sauletėkio Avn., 11, 10223 Vilnius, Lithuania; 2 Laboratory of Experimental Mechanics, Institute of Mechanical Science, Faculty of Mechanics, Vilnius Gediminas Technical University, Basanavičiaus Str., 28, 03224 Vilnius, Lithuania; arturas.kilikevicius@vilniustech.lt; 3 Department of Information Systems, Faculty of Fundamental Sciences, Vilnius Gediminas Technical University, Sauletėkio Avn., 11, 10223 Vilnius, Lithuania; dalius.mazeika@vilniustech.lt

**Keywords:** piezoelectric energy harvester, bi-directional energy harvester, multi-modal piezoelectric energy harvester, cantilever array

## Abstract

The paper presents numerical and experimental investigations on a bi-directional multi-modal energy harvester which is based on a piezoelectric saw-tooth cantilever array. The harvester is composed of four piezoelectric cantilevers which are connected rigidly to each other. At each junction of the cantilevers, there are placed seismic masses which are used to reduce resonant frequencies of the cantilever array. Moreover, at the center of the cantilever array is placed a Z-shaped seismic mass, which is used to obtain an additional rotation moment during excitation of the energy harvester to this way increase the stability of output characteristics via the whole angular range. The rigid connection between cantilevers ensures the transfer of bending deformations from cantilevers which are resonant to cantilevers which are out of resonance operation mode. The design of cantilever array ensures that all piezo ceramics are affected or partly affected by bending deformations while excitation frequency changes from 10 Hz to 160 Hz. In addition, such a composition of the array ensures the multi-modal operation principle. Additionally, the proposed cantilever array is designed to respond to changes of excitation force angle in an XY plane. The numerical and experimental investigation have shown that the proposed energy harvester has four resonant frequencies at a range from 10 Hz to 160 Hz. The electrical characteristics of the harvester were investigated as well. The results of these investigations have shown that cantilever array is able to provide an average output power of 15.3 mW while excitation amplitude is 0.5 m/s^2^ and the angle of excitation force changes in range from 0° to 350°.

## 1. Introduction

Application of low-power, wireless electronics, wireless sensor nodes and internet of things (IoT) systems in industry and everyday life have changed our understanding of electronic systems mobility, application flexibility as well as having expanded monitoring, control and data collection possibilities [1,2]. However, these systems, in most cases, have a power supply based on conventional electrochemical batteries [3]. Application of the batteries in these systems has one main disadvantage—it must be replaced or recharged periodically. Considering that, in most cases, wireless systems are used in hard-to-reach areas or in mobile monitoring devices, recharge or replacement of the batteries, during operation of the system, ensures additional costs for maintenance which are, in most cases, higher than the cost of a sensor node or even the whole wireless monitoring or data collection system. Therefore, in order to increase intervals of periodical batteries’ recharging and replacement, or even to eliminate it, ambient energy harvesting technologies must be incorporated into wireless systems to recharge or replace conventional batteries [4,5,6]. Therefore, during the last decade, huge attention was placed into the development of low-power ambient energy harvesting technologies. For now, the most promising is vibration energy harvesting via different types of energy conversion principles such as: electrostatic, electromagnetic, tribological and piezoelectric. Electrostatic energy harvesters have a demand on external high-voltage battery as well as, in most cases, having complex designs. On the other hand, electromagnetic vibration energy harvesters are based on magnetic fields which are harmful for electronic devices and as a result cannot be mounted close to them. Moreover, electromagnetic harvesters are sensitive for external magnetic fields which can affect operation and output performance. Finally, tribological vibration energy harvesters have very poor output characteristics and low reliability [7,8,9,10,11,12]. On the other hand, piezoelectric low-power vibration energy harvesters do not have a demand on the external battery, are magnetic field free, are simple in design and are able to provide suitable output characteristics to drive the electronic systems or recharge batteries [13].

Piezoelectric vibration energy harvesters, in most cases, are based on piezoelectric bimorph cantilevers. Usage of such harvester design leads to two main disadvantages. Firstly, effective operation of the harvester is limited by a narrow frequency range, which allows the use of the harvester efficiently with several hertz around the resonant frequency of the cantilever. However, if the frequency of input vibrations changes more than several hertz, the output characteristics of the harvester fall down drastically [14,15,16,17]. In order to solve these disadvantages, we introduced several solutions and techniques: usage of cantilever array, usage of bi-stable or non-linear operation principle, manual or automatic frequency tuning techniques via mechanical or electrical interaction and the multi-modal operation principle. The usage of these techniques and methods allows to partly or fully eliminate the dependency of the piezoelectric energy harvester’s performance to the frequency of input vibrations [18,19,20,21,22]. The second disadvantage of the piezoelectric vibration energy harvester, which is based on a single cantilever, is dependence on the direction of input vibrations, i.e., in most cases, the energy harvesters are designed to capture vibrations which are in line with or normal to the main axis of the energy harvester. However, if the angle of vibration motion changes or the orientation of the energy harvester mismatches the motion direction, the output characteristics will drop down [23,24,25]. Therefore, in order to overcome these disadvantages, energy harvesters must be suitable to operate in the wider frequency range and capture vibrations which changes motion direction dynamically. The best balance between these demands is able to provide multi-modal bi-directional piezoelectric energy harvesters [26].

Wei et al. introduced a tower-shaped three-dimensional piezoelectric energy harvester based on three curved piezoelectric beams which are distributed radially, while the seismic mass for all cantilevers was the same [27]. In accordance with the numerical and experimental investigations, the harvester is able to capture vibrations with motions aligned to in-plane and out-of-plane directions and provide up to 65.8 µW and 17.2 µW of output power, respectively. On the other hand, considering the report, the energy harvester is not able to capture the vibrations which are not aligned to in-plane or out-of-plane directions. Therefore, the harvester has limited angle response. In addition, the harvester has two resonant frequencies, i.e., 2.8 Hz and 11.5 Hz. As a result, the harvester covers a very narrow frequency range via the multi-modal operation principle and does not provide the possibility to use it in areas with wider frequency spectrum of input vibrations.

Chen et al., reported on an M-shaped tri-directional piezoelectric energy harvester [28]. The harvester is based on an M-shaped structure suitable to respond to the vibrations with three different motion directions. The tri-directional response to vibrations of the harvester is based on different bending and torsional vibration modes of the M-shaped structure. Experimental investigations of the energy harvester showed that it is able to provide 76.12 µW, 84.72 µW and 125.07 µW of output power while vibrations are aligned in vertical, lateral and horizontal directions, respectively. Considering the claimed tri-directional operation principle of the harvester, it can be noticed that it can be obtained only if vibration frequency and direction are in coincidence with resonant frequency and modal shape. Moreover, the harvester does not exhibit the multi-modal operation principle. Therefore, the proposed energy harvester can be applied only for vibrations with particular frequency and motion directions. Due to this, the harvester’s application becomes complicated and restricted by special requirements to vibrations and their motion directions as well as frequencies.

Sun et al. reported on a multi-directional piezoelectric energy harvester with a frequency tuning function and the multi-modal operation principle [29]. The harvester is based on four cantilevers mechanically coupled via a bridge, which is compressively buckled via beams with Kurigami cuts. The frequency tuning is implemented via changes of the buckling force while the multi-modal and multi-directional operation principle obtained is via the usage of several deformation modes of the harvester, i.e., via usage of six different vibration modes. Numerical and experimental investigations of the structure show that frequency and angle response as well as electrical characteristics are promising. However, multi-directional response is obtained via different deformation modes of the structure, i.e., the motion of vibrations must be aligned to one of the axis of the harvester and in coincidence with one of deformation modes of the structure.

The manuscript presents numerical and experimental investigations of a bi-directional multi-modal saw-tooth piezoelectric cantilever array. The proposed energy harvester is composed of three cantilevers, which are composed of one indissoluble structure via rigid junctions between cantilevers while several seismic masses are used to reduce resonant frequencies and increase the performance of the harvester. The usage of several cantilevers ensures multi-modal operation principle while composition of cantilevers to one structure and usage of seismic masses ensures the induction of stresses in the whole structure during resonance of one of cantilever. The bi-directional operation principle in an XY plane of the proposed energy harvester is obtained via a saw-tooth shape. The saw-tooth shape ensures that the energy harvester acts as a spring with seismic mass placed on it, and through this, responses to the vibrations with different motion directions are obtained. In comparison to state-of-art harvesters, the proposed energy harvester has the following advantages: bi-directional operation principle is not linked or related to vibration modes of the structure, i.e., the harvester’s ability to capture input vibrations which change motion direction in time domain is not limited or restricted by particular vibration mode and it modal frequency. The composition and design of energy harvester ensures multi-modal operation principle which leads to concentration of several resonance frequencies and narrow frequency range and by this way ensures more flexible application of energy harvester as well as reduces dependence of output characteristics to the frequency of input vibrations. Finally, composition of bi-directional and multi-modal operation principles ensures proper harvester operation while the angle and frequency of input vibrations changes dynamically, i.e., the harvesters provide the possibility of obtaining relativity stable output characteristics while the frequency and angle of input vibrations have unsystematic and unpredictable changes in the desired angular and frequency domains.

## 2. Design and Operation Principle

**Figure 1:**
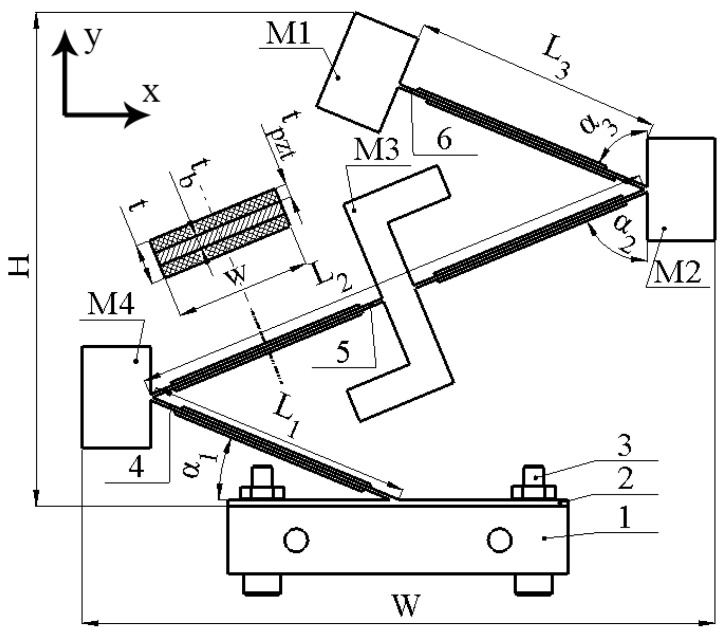
Sketch of bi-directional multi-modal piezoelectric energy harvester; 1—clamping base; 2—clamping plate of harvester; 3—clamping bolts of harvester; 4—6 piezoelectric cantilevers; M1–M4 seismic masses.

The energy harvester is based on three piezoelectric bimorph cantilevers with different lengths. The passive layer of cantilevers made from beryllium bronze C17200, while piezo ceramic plates are made from PIC 255 (PI Creamics, Lederhose, Germany). So, the cantilevers are composed of one structure via indissoluble rigid junctions and forms the saw-tooth-shaped body of the harvester. In addition, the harvester consists of three seismic masses, which are located at each junction and free ends of the cantilevers. Moreover, at the center of the second cantilever is placed a Z-shaped seismic mass which is used to induct an additional rotation moment of the whole structure and through this increases the stability of the electrical characteristics via the whole angular range. Clamping of the harvester is based on a passive beam, which is fixed to the vibration source via two bolts. The sketch of the energy harvester and dimensions of it are given in Figure 1 and Table 1, respectively.

**Figure 2:**
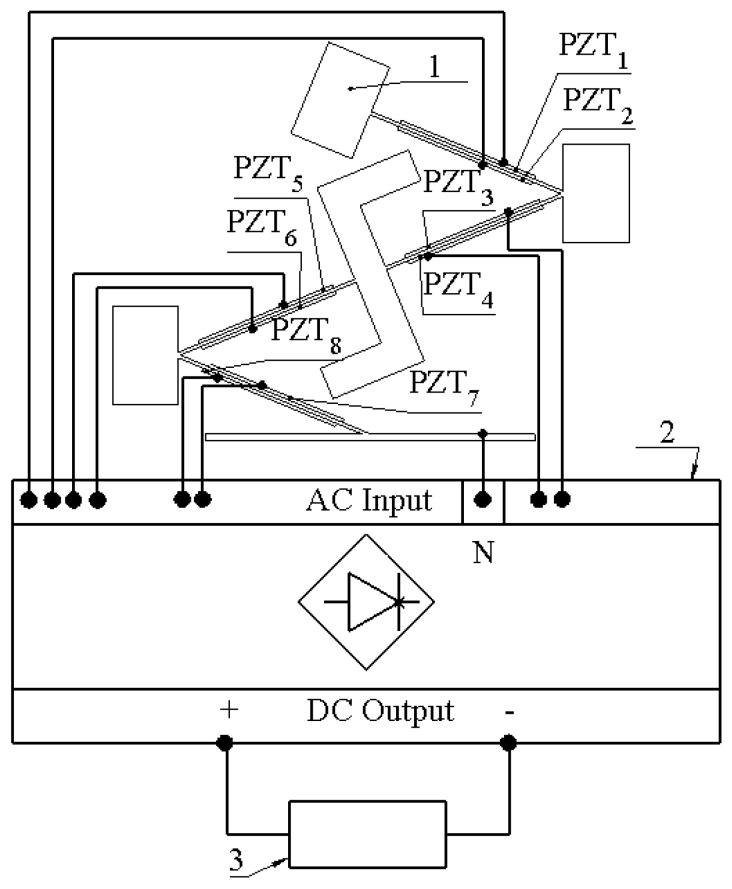
Basic electrical diagram of electrical connections; 1—the energy harvester; 2—multi-channel AC/DC converter; 3—resistive load; PZT_1_–PZT_8_—piezoelectric ceramics.

As can be found in Figure 1 and Figure 2, the energy harvester is composed of a saw-tooth shaped cantilever array with seismic masses. Such a composition of cantilevers ensures that the structure will act as a spring from which stiffness, in the in-plane direction, is lower compared to the out-of-plane direction. So, composition of the cantilevers to one rigid saw-tooth cantilever array ensures that the structure will act as a spring with anisotropic stiffness and ensures the harvesters’ response to any in-plane vibration, i.e., it becomes a bi-directional energy harvester. Moreover, the composition of the cantilevers to saw tooth shaped array ensures not only a bi-directional, but also a multi-modal operation principle, i.e., several cantilevers with similar geometrical and mechanical characteristics will ensure several resonance frequencies at a narrow frequency range. In addition, the composition of the cantilever array to one rigid structure ensures partial strain transfer from active cantilevers to passive, i.e., during resonance of one part of the cantilever array, passive parts are fully or partly affected by inducted strain via reaction forces generated by the resonating part. Finally, seismic masses, M1, M2 and M4 are used to reduce resonant frequencies of the energy harvester structure and to improve output characteristics via higher strain generation while Z-shaped (M3) seismic mass was designed to induct an additional rotation moment of the whole structure and as a result via the shifted center of the mass amplify strain generated by it.

As can be found in Figure 1 and Figure 2, the energy harvester is composed of four cantilevers, which are covered by eight piezo ceramics. The width of piezo ceramic plates, used to compose the energy harvester, are equal to the width of the passive layer (W), while lengths of the piezo ceramic plates are shorter by 30% compared to the length of the cantilevers (Figure 1, Table 1). The coverage of cantilevers by piezo ceramic plates is 70%. Such coverage is affected by the design of whole structure, i.e., sharp edges between cantilevers which limits full coverage of the structure. The capacity of piezo ceramic varies from 6.7 nF to 7.1 nF while the total capacitance is around 55.1 nF. In order to avoid possible charge cancelation phenomena, during the harvester’s operation at different deformation modes, especially at the second bending mode, each piezo ceramic was connected to a multi-channel AC/DC converter, which was based on Schotty diodes (BAT48, STMicroelectronics, Switzerland). The AC/DC converter is based on eight full diode bridge rectifiers. Each rectifier was dedicated for a particular piezo ceramic plate, i.e., the neutral wire of the energy harvester was connected to the common AC input (Figure 2, COM), while the hot wires of each piezo ceramic plate were connected to a particular diode bridge. Such a connection allows the prevention of charge cancelation phenomena during the harvester’s operation at different deformation modes. Finally, positive and negative DC outputs of each diode bridges were connected in parallel in order to obtain common output of the energy harvester. Therefore, such a design of a multi-channel AC/DC converter proposes the possibility to connect several piezo ceramic plates, which generates charges with different polarities and obtains a common DC output. A basic diagram of the electrical connections is given in Figure 2.

## 3. Numerical Investigation of an Energy Harvester

In order to perform numerical investigation, the finite element model (FEM) of the energy harvester was built by Comsol Multiphysics 5.4. The geometrical parameters were set with strict adherence to Table 1 and Figure 1, while material characteristics were set in accordance with Table 2.

**Figure 3:**
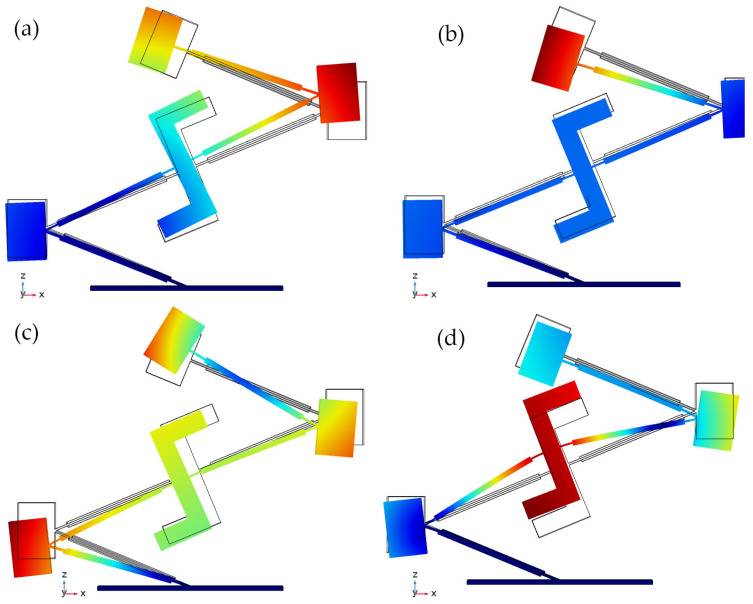
Modal shapes of energy harvester; (**a**)—at 18.19 Hz; (**b**)—at 49.57 Hz; (**c**)—at 76.31 Hz; (**d**)—at 170.77 Hz.

The first stage of numerical study was dedicated to modal analysis of the energy harvester. For this study, the electrodes of piezo ceramic plates were set to a short circuit condition, the in-plane orientation of the harvester was aligned to the *z* axis, the input vibration amplitude was set to zero, while the base of the harvester was fixed rigidly. The results of the calculations are given in Figure 3.

As can be found in Figure 3, the energy harvester has four vibration modes at a range from 10 Hz to 180 Hz which are based on one bending mode of the cantilevers or a compound of several bending modes of several cantilevers. In addition, it can be found that all vibration modes are in an in-plane direction. Therefore, these modes are suitable for energy harvester operation in a bi-directional multi-modal regime. Moreover, it can be found that vibration modes ensure the vibrations of a whole or part of the structure. Therefore, piezo ceramics on passive cantilevers will be, fully of partly, affected by stresses inducted by active cantilevers.

**Figure 4:**
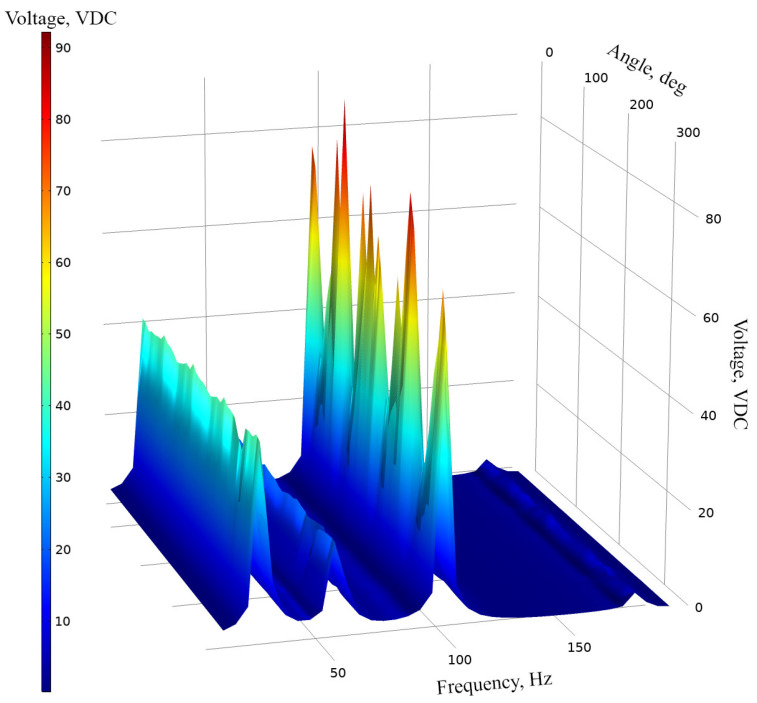
Open-circuit voltage characteristic of energy harvester.

The next stage of numerical investigation was dedicated to the calculation of open-circuit voltage (OCV) characteristics. An AC/DC converter, based on eight diode bridge rectifiers, was included in the model in order to prevent possible charge cancelation phenomena during the harvester’s operation at different deformation modes. Each diode bridge rectifier was dedicated to a specific piezo ceramic, while outputs of rectifiers were connected in parallel in order to obtain common output voltage characteristics. Output resistance of the AC/DC converter was set to an open circuit condition. Acceleration of input vibrations was set to 0.5 m/s^2^, and the input vibrations were applied to the base of the energy harvester while analyzed, and the frequency range was set from 10 Hz to 200 Hz with step resolution of 5 Hz. Moreover, the angle of excitation motion was set to range from 0° to 350° in the XZ plane with a step resolution of 10°. The results of the calculations are given in Figure 4.

**Figure 5:**
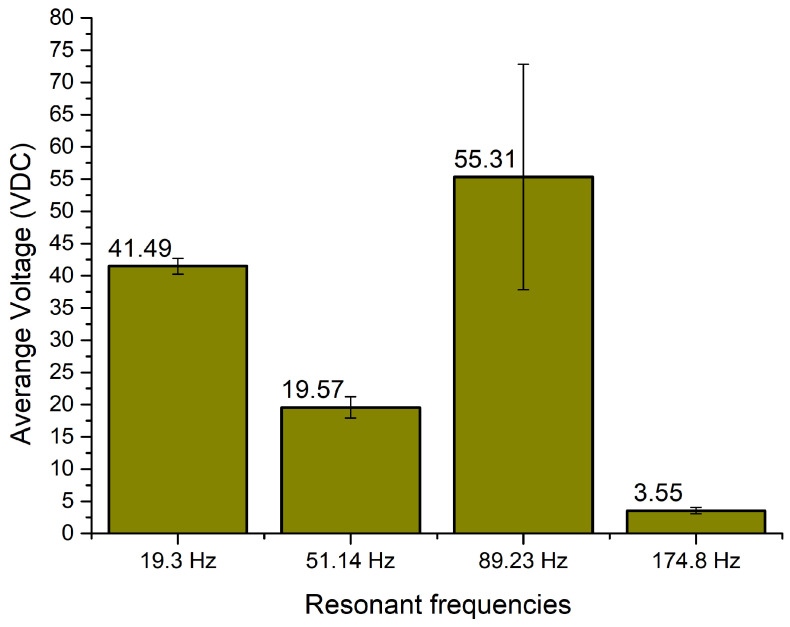
Summary of average open-circuit voltage over angular range.

As can be found in Figure 4, OCV characteristics of the energy harvester confirms the results of modal analysis and shows that the indicated vibration modes are suitable for bi-directional, multi-modal operation. Additionally, the characteristics show that the energy harvester is able to propose electrical characteristics while the angle of excitation vibrations changes from 0 to 350 degrees. Compared to other resonance frequencies, at the third resonance frequency there can be observed more notable fluctuations of OCV values. Considering that the shape of the third vibration mode involves the whole structure of the energy harvester, vibration damping occurs which is influenced by changes and differences of the harvester’s displacement phases at different angles. In addition, reaction forces which are generated by seismic masses, at different angles, also plays a notable role in the damping of the energy harvester, while the third resonance is excited. Therefore, the fluctuations are notable and will have influence in the deviation of the average OCV value or other electrical characteristics. In order to analyze the results of calculations, the summary of average OCV values, at resonance frequencies, was made. The average values were calculated for whole angular range of vibrations. The summary is given in Figure 5.

As can be found in Figure 5, resonance frequencies have slight differences compared to modal frequencies which were obtained during modal study. The differences do not exceed 14.47%. The differences in frequency values occurred due to differences in electrical boundary conditions during modal and frequency response analyses.

The summary shows that the highest average open-circuit voltage obtained at the third resonance frequency and reached 55.31 VDC. However, at this frequency, the standard deviation of the value also has the highest value, i.e., 17.49 VDC. The lowest average open-circuit voltage obtained at the fourth resonance and reached 3.55 VDC with minor, 0.476 VDC, deviation over the whole angular range. Similar tendencies can be observed in characteristics of the first and second resonances, i.e., 41.49 VDC with deviation of 1.23 VDC and 19.57 VDC with deviation of 1.64 VDC, respectively.

Therefore, on the basis of these results, it can be assumed that the energy harvester will be able to provide electrical characteristics during changes in angle of input vibrations in spite of the deviation of the average open-circuit voltage at the third resonance being at a relatively high level.

**Figure 6:**
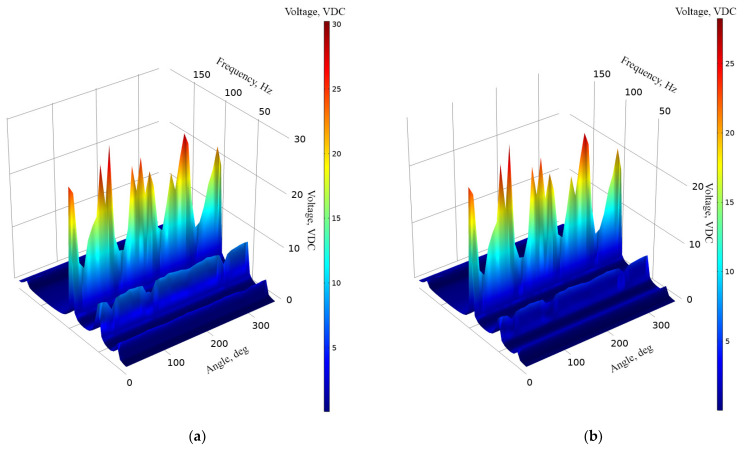

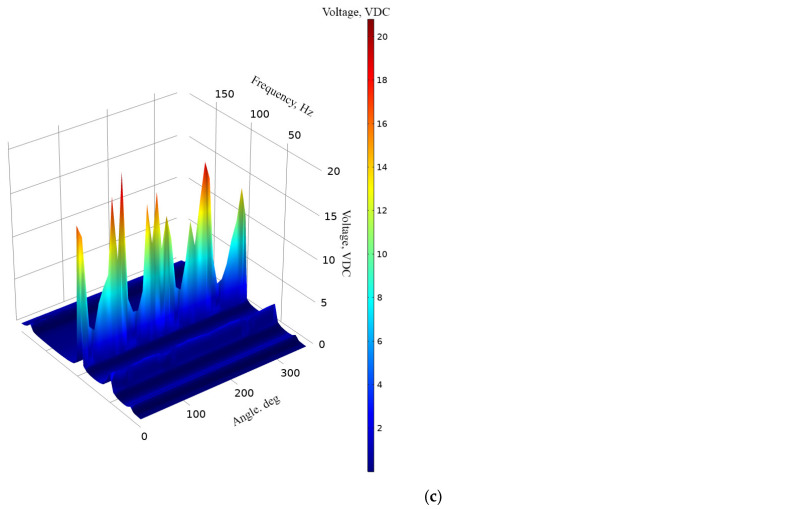
Frequency—voltage—angle of excitation characteristics of the energy harvester with different loads; (**a**)—load equal to 1 MΩ; (**b**)—load equal to 500 kΩ; (**c**)—load equal to 250 kΩ.

The next stage of numerical investigation was performed in order to indicate the energy harvester’s frequency—voltage—and the angle of vibration motion characteristics under different resistive loads. The values of resistive loads were set to 1 MΩ, 500 kΩ and 250 kΩ. Studies for each resistive load case were performed separately. The amplitude of input vibrations was set to 0.5 m/s^2^, while the range of the angle of input vibrations was set from 0° to 350° with step of 10°. The results of the calculations are given in Figure 6.

As can be found in Figure 6, frequency—voltage—and the angle of excitation motion direction characteristics confirm the results of modal analysis as well as the calculations of the OCV characteristics. Despite the resistive load value, the characteristics contain four resonance frequencies and which shows that the energy harvester is able to provide the multi-modal operation principle while the angle of excitation motion changes. In addition, it can be found that the characteristics are stable over angle changes; only the third resonance frequency has notable fluctuations which are the result of modal shape response to the angle of excitation motion.

**Figure 7:**
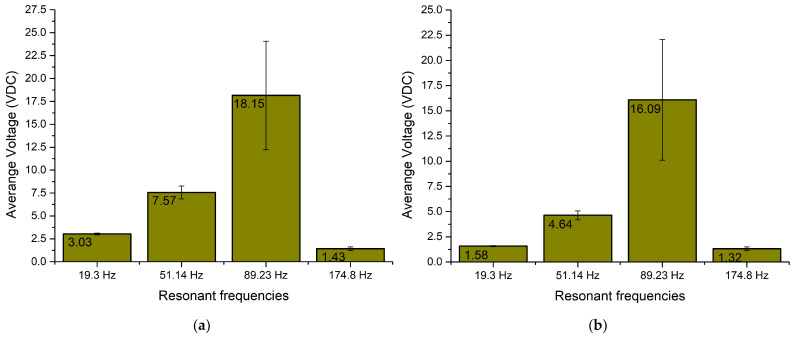

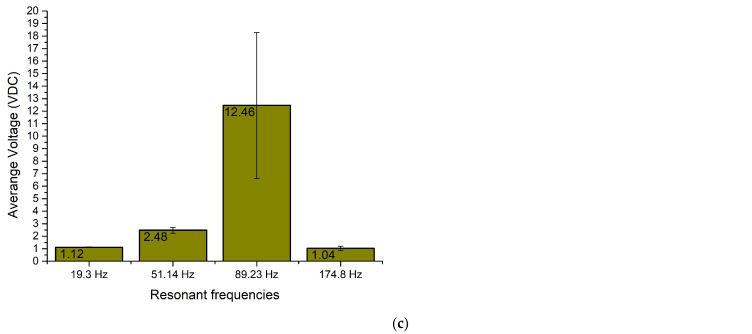
Summary of average output voltages at resonance frequencies while different loads are used; (**a**)—load equal to 1 MΩ; (**b**)—load equal to 500 kΩ; (**c**)—load equal to 250 kΩ.

Figure 6a represents the characteristics of an energy harvester while the resistive load is equal to 1 MΩ. The highest voltage value was obtained while the frequency was equal to 89.23 Hz and reached 30.25 VDC. On the other hand, the lowest output voltage was obtained at the fourth resonance frequency, i.e., 174.8 Hz and reached 1.85 VDC. Figure 6b shows that the highest output voltage, while the load was equal to 500 kΩ, reached 28.26 VDC at 89.23 Hz. It can be found that the value has a minor difference compared to the case with 1 MΩ, i.e., only 1.99 VDC. However, it is worth considering that in the graphs it can be found that the fluctuations are notably higher while a 500 kΩ load is applied. It is the influence of the increased damping of the energy harvester which occurs due to a lower resistive load. The lowest output voltage at a 250 kΩ load was obtained at a fourth resonance frequency and reached 1.73 VDC. Finally, in Figure 6c, it can be found that the highest output voltage reached 25.01 VDC at the third resonance frequency. At the same resistive load, the lowest output voltage reached 1.42 VDC and it was obtained at the fourth resonance frequency. On the other hand, these values, which were obtained at different loads, represent the maximum values via the whole range of angles. In order to fully represent the harvester’s characteristics, a summary of average output voltages at all resonance frequencies was made. Additionally, the standard deviation of the values was calculated. The summary is given in Figure 7.

As can be found in Figure 7, the average voltage values at the first, second and fourth resonance frequencies are stable and have low deviations. On the other hand, the average output voltage value obtained at the third resonance has a notable deviation. In general, it is influenced by the modal shape of energy, and it responds to changes in the angle of input vibration. At particular angles of excitation motion the harvester resonates partly and does not generate the full amount of possible output voltage.

Additionally, considering Figure 6 and Figure 7, the third resonance, despite deviations, ensures the highest output voltage. It is the result of higher employment of the harvester’s body which is achieved via its design and the transfer of stresses between different parts of the energy harvester. On the other hand, the average output values of voltages at other resonances are lower but stable over all ranges of the angles of excitation motion.

**Figure 8:**
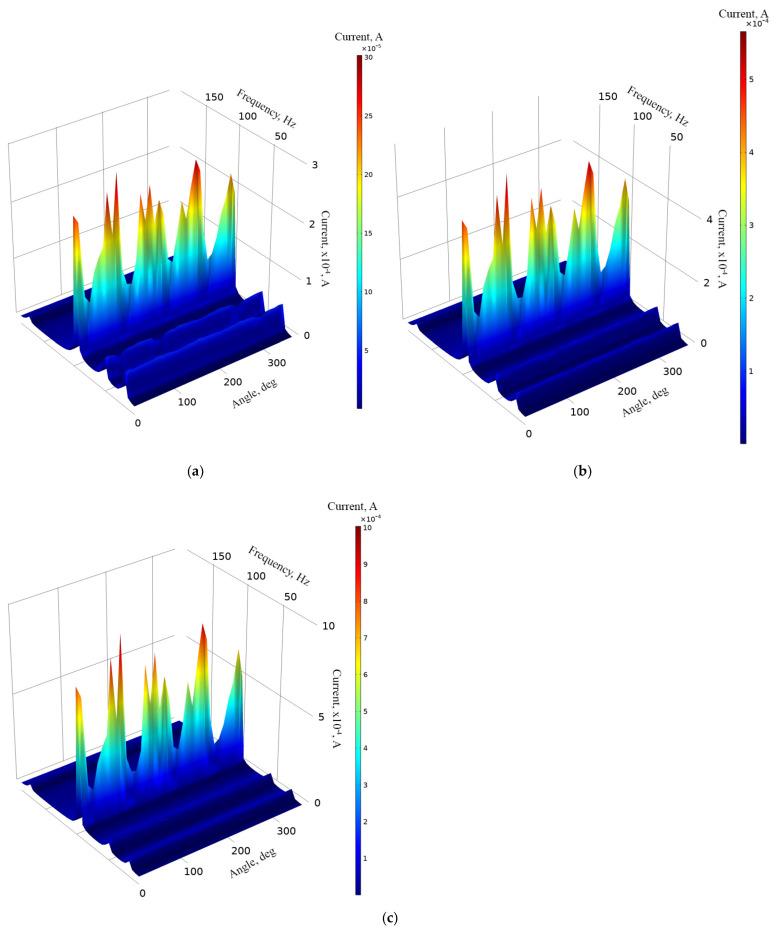
Frequency—current—angle of excitation motion characteristics of the energy harvester with different loads; (**a**)—load equal to 1 MΩ; (**b**)—load equal to 500 kΩ; (**c**)—load equal to 250 kΩ.

The next stage of numerical investigation was dedicated to the calculation of the output current while different resistive loads are connected as well as angle of excitation motion changes from 0° to 350° with a step of 10°. The calculations were made with the same boundary conditions as in the case before. The results are given in Figure 8.

As can be found in Figure 8a, the maximum output current was obtained at the third resonance and reached 301.9 μA while the resistive load was 1 MΩ. However, it can be found that there occurs a notable fluctuation over angle of excitation motion. On the other hand, the lowest output current was obtained at the fourth resonance, and it reached 4.37 μA at the same load.

Figure 8b represents the frequency—current— and the angle of excitation motion characteristics while a 500 kΩ resistive load was connected to the common output of the energy harvester. The highest output current reached 566.21 μA at the third resonance while the lowest output current value was obtained at the fourth resonance and reached 28.48 μA. Considering Figure 8c, it can be found that the highest output current reached 1.04 mA while a 250 kΩ load was connected. The lowest output current with this load was obtained at the fourth resonance frequency and reached 46.44 μA.

**Figure 9:**
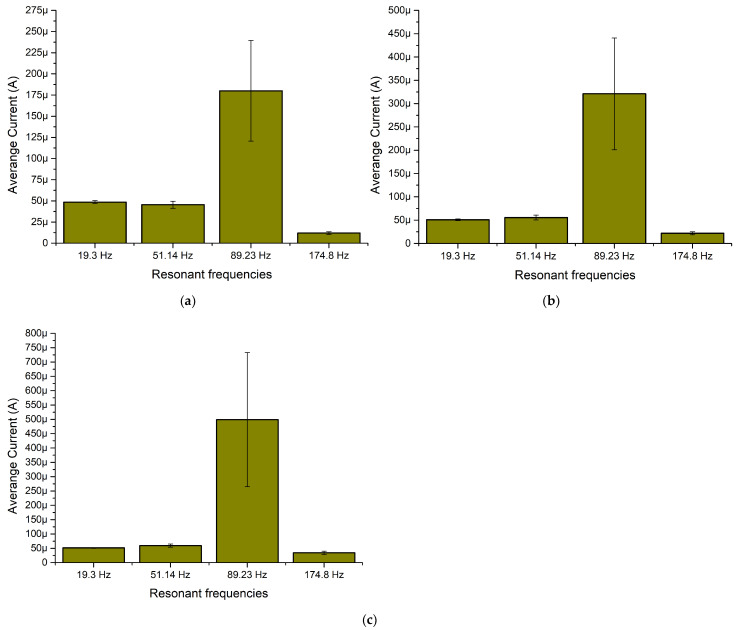
Summary of average output current at resonance frequencies while different loads are used; (**a**)—load equal to 1 MΩ; (**b**)—load equal to 500 kΩ; (**c**)—load equal to 250 kΩ.

However, the maximum values which were introduced before, do not fully represents characteristics of an energy harvester. Therefore, in order to fully represent the frequency—current—and the angle of excitation motion characteristics, summary of average current values was made. The average values were calculated for each resonance frequency over the angle of excitation motion as well as the deviation of values. The summary is given in Figure 9.

As can be found in Figure 9, the lowest average output current is obtained at the fourth resonance frequency for all load cases. However, the deviation of output current values at the whole angular range is minor. In addition, compared to the fourth resonance, output currents at the first and second resonance frequencies are notably higher and also have low deviations over the whole angular range. Therefore, it can be found that the energy harvester will be able to provide stable output characteristics despite the angle of excitation motion. On the other hand, the highest average output current was obtained at the third resonance. The value is notably higher compared to the characteristics obtained at other resonances. However, the deviation is high and the output current characteristics at this frequency are less stable and more sensitive to the angle of excitation motion.

**Figure 10:**
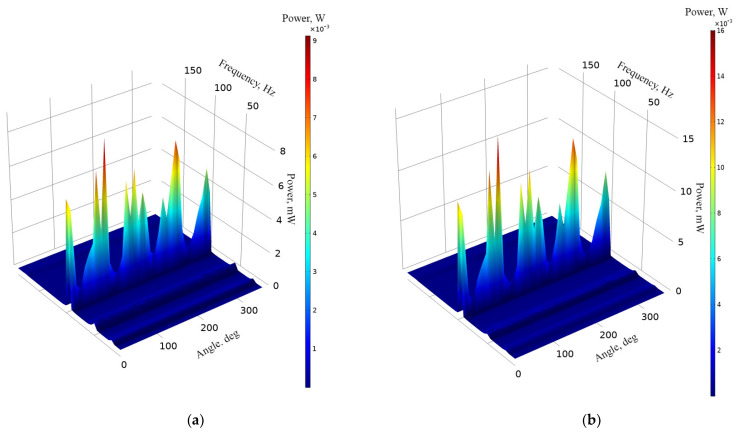

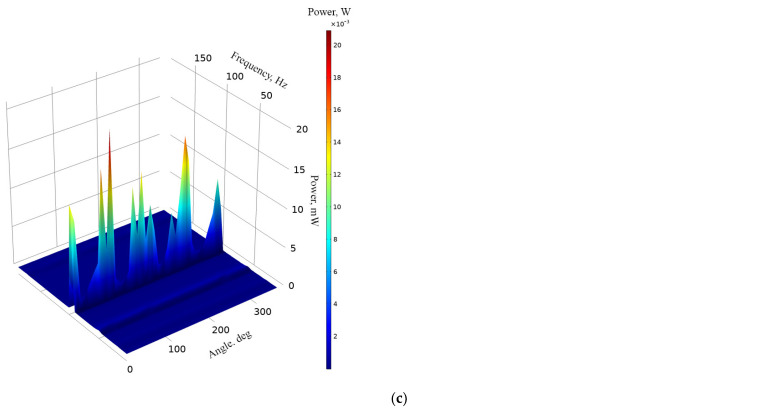
Frequency—power—and angle of excitation motion characteristics of the energy harvester with different loads; (**a**)—load equal to 1 MΩ; (**b**)—load equal to 500 kΩ; (**c**)—load equal to 250 kΩ.

The next stage of numerical investigation was dedicated to the calculation of the energy harvester’s output power characteristics. The calculations were made at the same mechanical and electrical boundary conditions as in cases before. The results are given in Figure 10.

**Figure 11:**
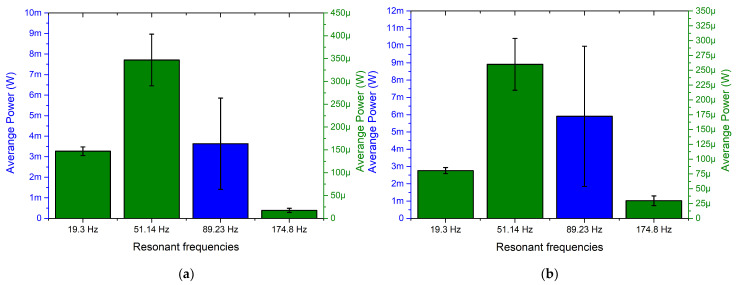

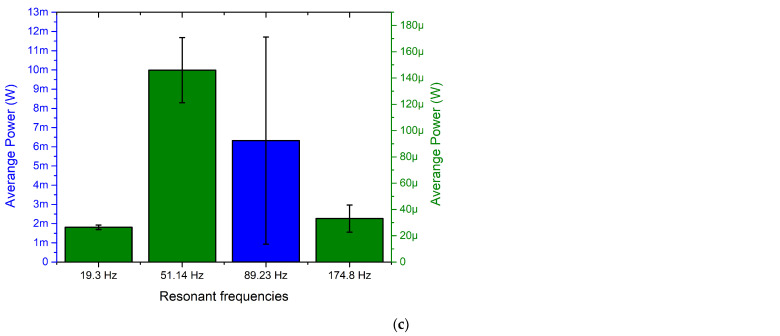
Summary of average output power at resonance frequencies while different loads are used; (**a**)—load equal to 1 MΩ; (**b**)—load equal to 500 kΩ; (**c**)—load equal to 250 kΩ.

As can be found in Figure 10, the highest output power values were obtained at the third resonance frequency at all load cases. The maximum values are 9.1 mW, 16.3 mW, 209.12 mW while the loads were 1 MΩ, 500 kΩ, 250 kΩ, respectively. However, the characteristics, given in Figure 10, show that fluctuations of output power at this resonance are high and sensitive to the angle of excitation motion. On the other hand, the energy harvester also provides output power at other resonance frequencies. The values are notably lower but stable and have low fluctuations over angular range. Therefore, in order to fully define characteristics of the energy harvester, a summary of average output power at resonance frequencies was made. The summary is given in Figure 11.

As can be found in Figure 11, the highest average output power characteristics were obtained at the third resonance frequency at all load cases. However, the deviation of these values is high. Therefore, at this resonance frequency, the output characteristics notably depend on the angle of excitation motion. On the other hand, output power characteristics at other resonance frequencies are much more stable and have a lower deviation over angular range. At these frequencies, the average output powers, compared to the third resonance, are several times lower. However, despite the deviations, it can be found that the energy harvester is able to provide medium stability electrical output characteristics via the whole angular range as well as to propose the multi-modal operation principle.

## 4. Experimental Investigation

Experimental investigations were performed in order to confirm the results of numerical investigations as well as to experimentally indicate the electrical characteristics of the energy harvester.

**Figure 12:**
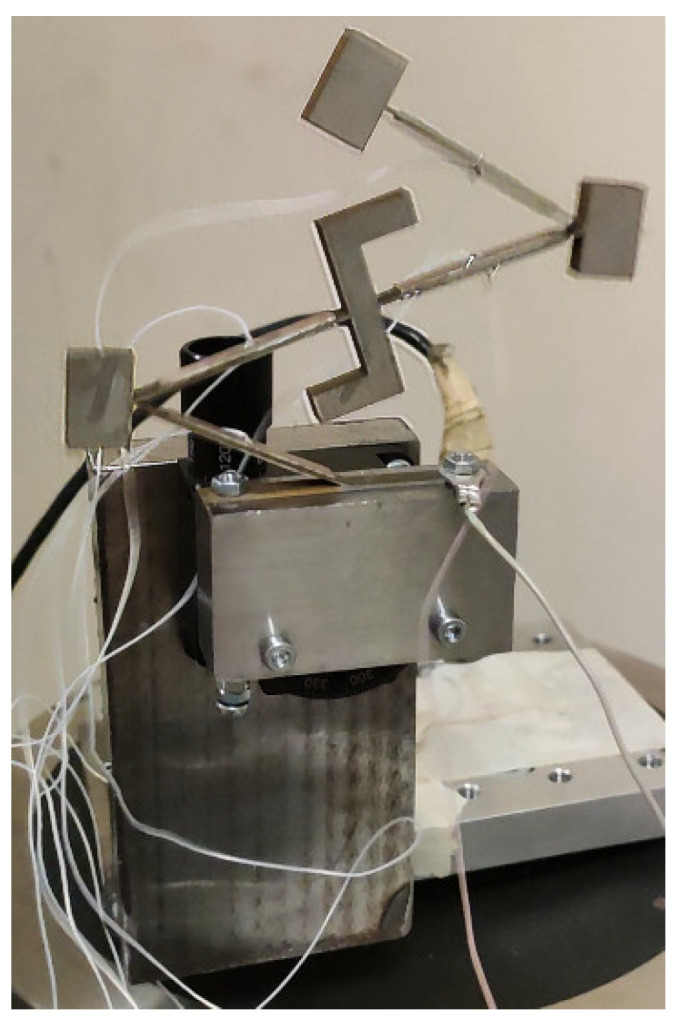
Prototype of the energy harvester.

For this purpose, the prototype of the energy harvester was made with strict respect to the geometrical characteristics which are indicated in Figure 1 and Table 1. Additionally, materials used to build the energy harvester were chosen with respect to Table 2. Furthermore, special clamping of the energy harvester was designed with the purpose to obtain a controllable angle of excitation motion. A view of the prototype and its clamping is given in Figure 12.

**Figure 13:**
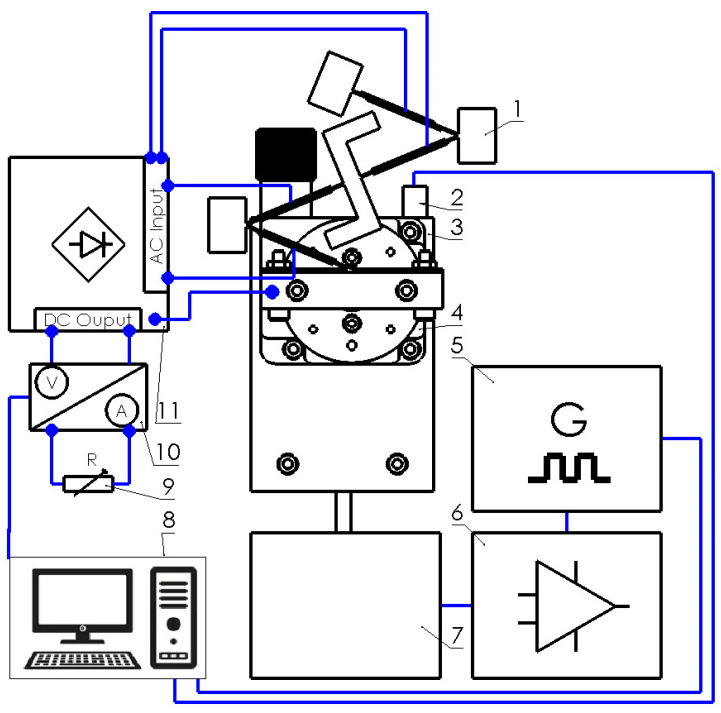
Experimental setup; 1—energy harvester, 2—accelerometer; 3—clamping of energy harvester; 4—rotatable table; 5—function generator; 6—power amplifier; 7—electromagnetic shaker; 8—computer; 9—variable resistor; 10—two channel multimeters; 11—AC/DC converter.

As can be found in Figure 12, the prototype is mounted on a special mounting system which consist of an L-shape body and rotatable table. Together with the mounting system, the energy harvester is fixed to an electromagnetic shaker. Such a mounting of an energy harvester ensures the possibility to control and change the angle of input vibrations. Experimental investigations were performed by employing an experimental setup. A schematic of this experimental setup is given in Figure 13.

As can be found in Figure 13, the experimental setup is based on an energy harvester and its clamping system with a rotatable table (Model 7R174-11, Standa, Vilnius, Lithuania) (Figure 13—1). The rotatable table is used to control the angle between input vibrations and the energy harvester. Therefore, the energy harvester, together with mounting system, is mounted on top of an electromagnetic shaker (Model 4806, HP, Palo Alto, CA, USA) (Figure 13—7) which is controlled by a computer via a functional generator (Model WW5064, Tabor Electronics, Tel Hanan, Israel) and a power amplifier (Type 2307, Brüel & Kjær, Nærum, Denmark) (Figure 13—6,5 and 8). The input vibrations are measured by an accelerometer (Type 4375, Brüel & Kjær, Nærum, Denmark) (Figure 13—2) which is connected to the computer. Therefore, the frequency and amplitude of vibrations are controlled by computer in parallel. Piezo ceramic plates (Figure 2, PZT_1_–PZT_8_) are separately connected to an AC/DC converter (Figure 13—11). The AC/DC converter is based on eight full wave diode rectifiers where outputs are connected in parallel in order to form a common DC output. Electrical characteristics are measured by two channel digital multimeter (Model WT300E, Yokogawa, Musashino, Tokyo, Japan) (Figure 13—10) with power measurement and data logging functions. The measured data is recorded and transferred to the computer. The electrical load of the energy harvester is implemented by a variable resistor (Figure 13—9) where the value is controlled manually.

**Figure 14:**
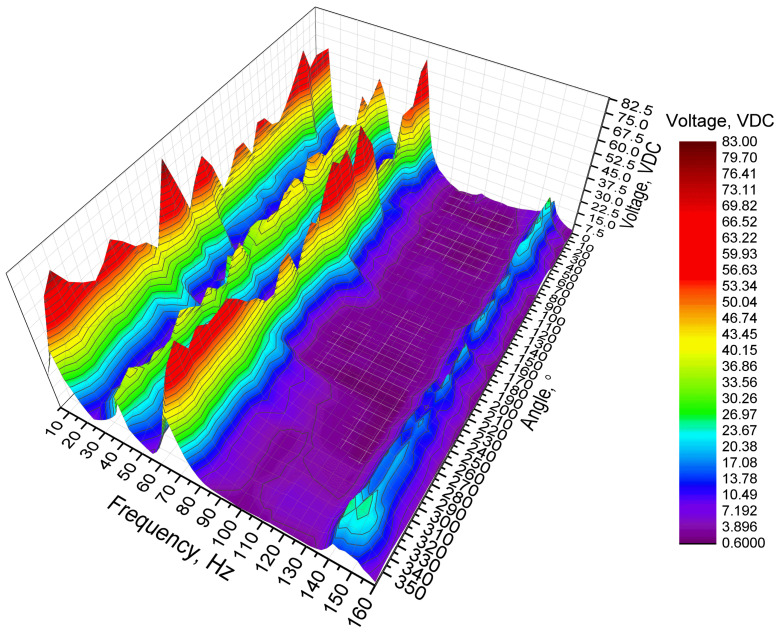
Open-circuit voltage characteristics of the energy harvester.

The first stage of experimental investigation was dedicated to measuring the OCV characteristics of the energy harvester. In order to perform the experimental investigation, the excitation frequency range was set from 10 Hz to 160 Hz with a step of 5 Hz. The excitation amplitude of the energy harvester was set to 0.5 m/s^2^ while the angular range was set from 0° to 350°. Finally, the value of the resistive load (Figure 13—9) was set to 25 MΩ, i.e., to an open-circuit condition. The results of the investigation are given in Figure 14.

**Figure 15:**
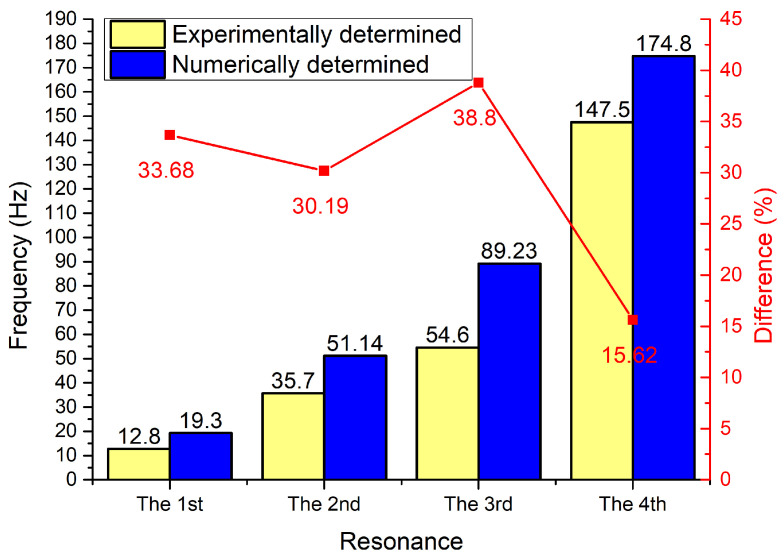
Comparison between calculated and experimentally determined resonance frequencies.

As can be found in Figure 14, the energy harvester has four resonance frequencies in a range from 10 Hz to 160 Hz. It confirms the results of modal analysis. However, resonance frequencies are lower compared to frequencies obtained during numerical investigation. The comparison between calculated and experimentally indicated resonance frequencies is given in Figure 15.

Figure 15 shows that experimentally determined resonance frequencies of energy harvesters are lower, i.e., differences vary from 15.62% to 38.8% compared to the result of numerical investigation. The main reasons of these differences are a mismatch in mechanical and electrical boundary conditions, differences in clamping conditions as well as manufacturing and assembly errors. However, considering that in the graphs it can be found that all resonance frequencies are lower. This aspect can be assessed as positive due to a better concentration of resonance frequencies at a narrower frequency range.

**Figure 16:**
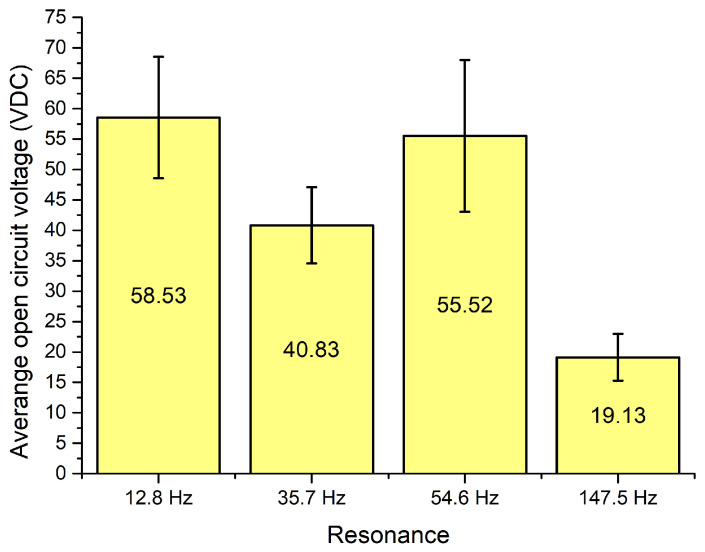
Summary of average open circuit voltage over angular range.

Additionally, during analysis of OCV characteristics (Figure 14) it was found that the highest output voltage was obtained at the first resonance frequency and it reached 82.87 VDC while the lowest OCV value was obtained at the fourth resonance and it reached 29.11 VDC. However, maximum values do not fully represent OCV characteristics of the energy harvester. Therefore, the average OVC values and their deviations were calculated at resonance frequencies via the whole angular range. The summary of these values is given in Figure 16.

Figure 16 shows that the highest average OCV value was obtained at the first resonance frequency and it reached 58.53 VDC, while at the third resonance it reached 55.52 VDC. Therefore, it can be found that at these frequencies, the average output characteristics are similar. On the other hand, the deviation of the value is higher at the third resonance. Therefore, the first resonance proposes more stable output characteristics compared to the third. On the other hand, the lowest average OCV characteristic was obtained at the fourth resonance ant it is three times lower compared to the highest value. However, the deviation of OCV at the fourth resonance is notably lower and it shows that the OCV characteristic at this frequency is more stable compared to the first resonance.

**Figure 17:**
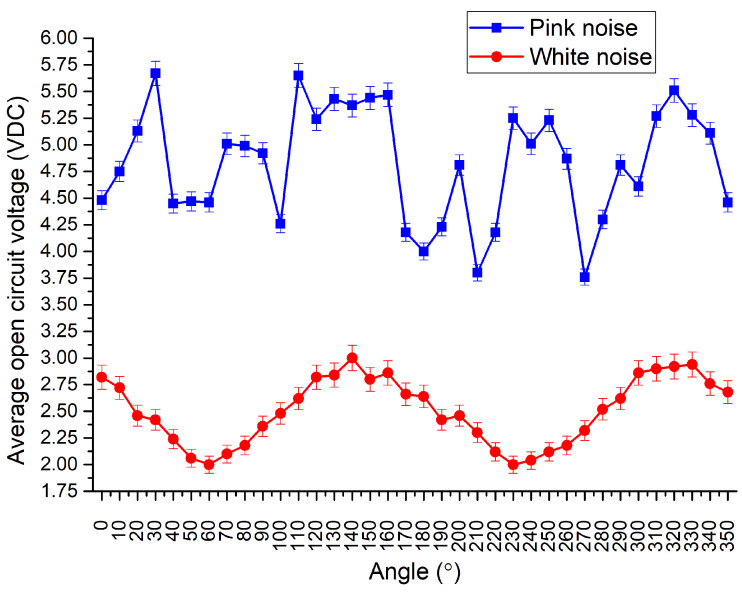
OCV—angle of excitation characteristics of energy harvester while input vibrations are random.

The next stage of experimental investigation was dedicated to the investigation of OCV characteristics while input motions were based on pink and white noise vibrations. The investigation was performed in order to indicate the harvester’s response characteristics to a random excitation signal which changes in the time domain. The results of the investigation are given in Figure 17.

As can be found in Figure 17, the harvester’s response to white noise has systematic behavior, i.e., the OVC changes with respect to the angle of input vibrations. Considering that white noise has a stable power spectrum over the frequency range, it can be found that the energy harvester’s response to white noise is directly related to the potential barrier of the structure which changes via the angular range. The average OCV value over the angular range is 2.5 VDC with a deviation of 0.31 VDC.

On the other hand, during the harvester’s excitation by pink noise vibrations, OCV characteristics are notably higher. The average OCV value is 4.82 VDC with a deviation of 0.52 VDC. Compared to the results obtained with vibrations based on white noise, the average output voltage is almost two times higher. It is the result of different input vibrations’ power via a frequency spectrum. However, the electrical characteristic over an angular range does not have systematic behavior. It is a result of a variable potential barrier of the energy harvester via angular range.

**Figure 18:**
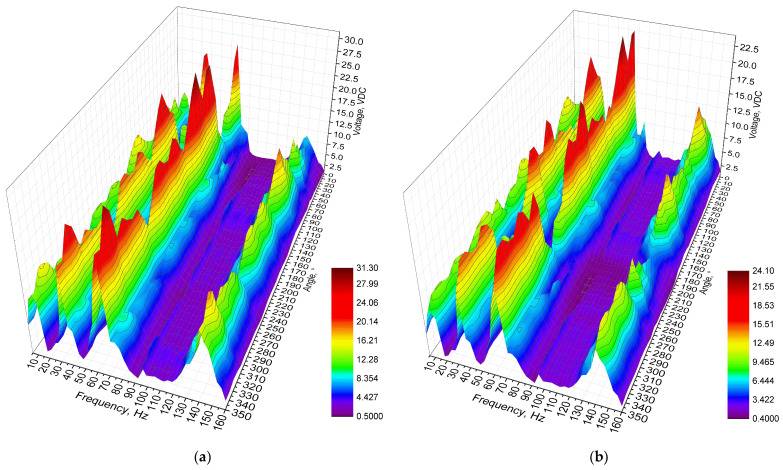

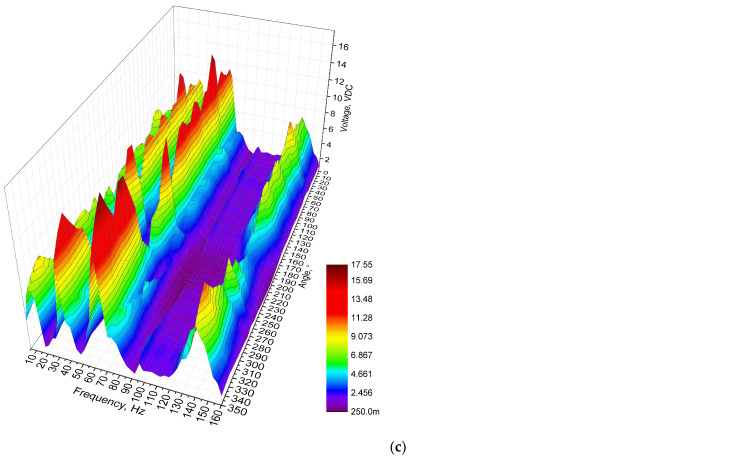
Frequency—voltage—and angle of excitation motion characteristics of the energy harvester with different loads; (**a**)—load equal to 1 MΩ; (**b**) —load equal to 500 kΩ; (**c**)—load equal to 250 kΩ.

Next stage of experimental investigation was dedicated to measurements of output voltage while resistive loads are connected to the energy harvester. Three different resistive loads were used for this investigation, i.e., 1 MΩ, 500 kΩ and 250 kΩ. Separate measurements were performed for each load case while the frequency range was set to range from 10 Hz to 160 Hz with a step of 2.5 Hz. In addition, input vibrations amplitude was set to 0.5 m/s^2^. The results of the measurements are given in Figure 18.

As can be found in Figure 18, the maximum output voltages were obtained at the third resonance frequency for all load cases. The maximum values reached 31.28 VDC, 24.08 VDC, and 17.51 VDC while the electrical loads were 1 MΩ, 500 kΩ and 250 kΩ, respectively. On the other hand, the lowest maximum values were obtained at the first resonance frequency and reached 16.91 VDC, 13.02 VDC and 9.47 VDC for the same electrical load cases. Therefore, it can be found that fluctuations of output values exist. However, compared to the results of numerical investigations, it can be noticed that the output voltages are notably higher, while fluctuations of the values are lower. It is the result of the usage of Z-shaped seismic mass, i.e., considering that the axis of excitation and the longitudinal axis of the energy harvester have a difference not only in the in-plane direction, but also in the out-of-plane direction where additional stresses occur which are influenced by the seismic mass with a shifted center of the mass. Therefore, due to this additional out of plane motion, generated due to a mismatch of axes, there occurs an additional rotation moment which notably affects the output characteristics of the energy harvester.

**Figure 19:**
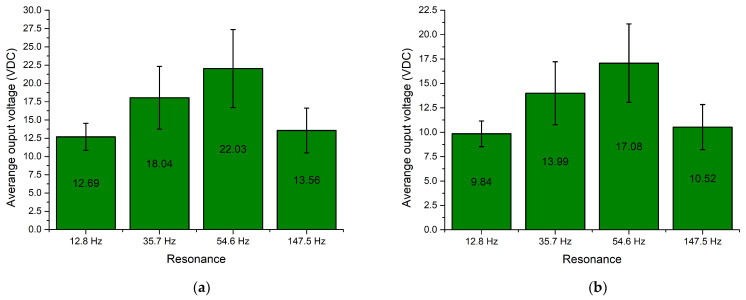

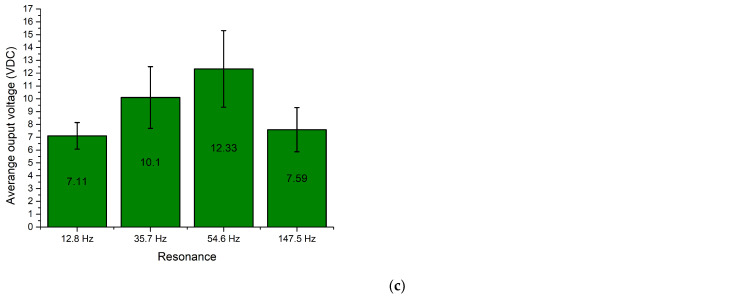
Summary of average output voltages at resonance frequencies while different loads are used; (**a**)—load equal to 1 MΩ; (**b**)—load equal to 500 kΩ; (**c**)—load equal to 250 kΩ.

In order to fully represent the characteristics of the energy harvester, the average output voltage values at resonance frequencies over the whole angular range were calculated, as well as the deviation of these values. The summary of characteristics is given in Figure 19.

As can be found in Figure 19, the highest average values of output voltage were obtained at the third resonance. The lowest average output voltages were obtained at the first resonance. However, the difference between the first and fourth resonances are minor. Therefore, compared to the results of numerical investigation, the trends of the characteristics are similar. Unfortunately, the deviation of the values is noticeable and has an effect on the stability of the output characteristics. Considering the deviation, the output voltage characteristics have a medium stability level. Therefore, it can be said that the energy harvester is able to provide suitable and relativity stable output voltage characteristics via the whole angular range.

**Figure 20:**
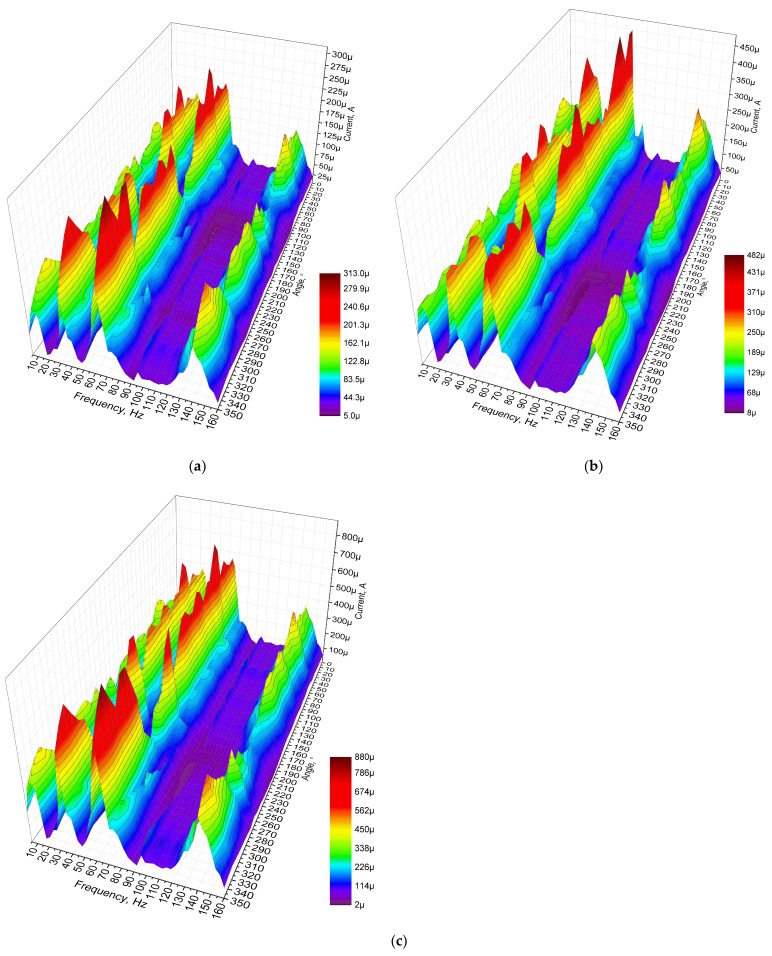
Frequency—current—angle of excitation motion characteristics of the energy harvester with different loads; (**a**)—load equal to 1 MΩ; (**b**)—load equal to 500 kΩ; (**c**)— load equal to 250 kΩ.

The next stage of experimental investigation was dedicated to measurements of the output current, while different resistive loads were used. The resistive loads, amplitude of input vibrations, frequency range, as well as angular range were set as in previous experimental investigation. The results of the investigation are given in Figure 20.

**Figure 21:**
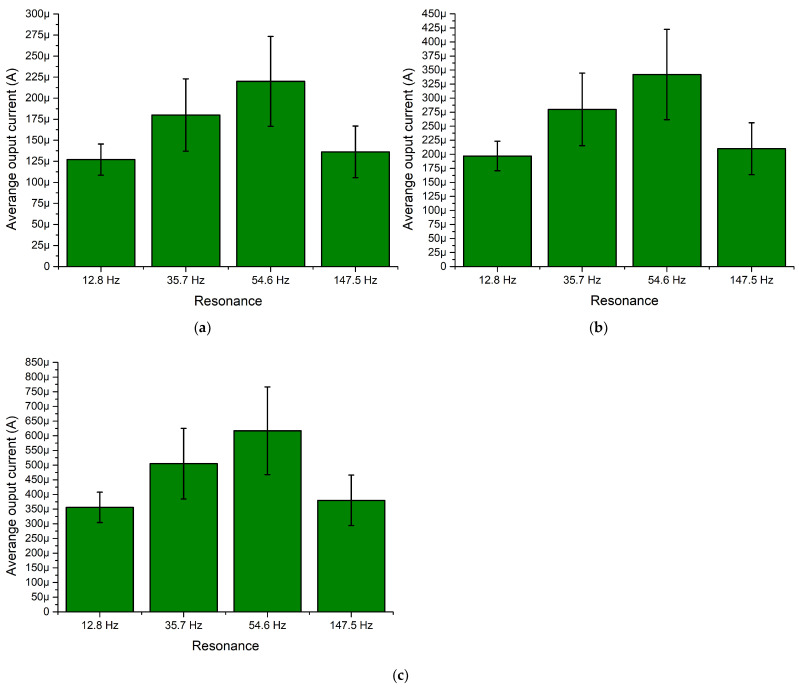
Summary of average output currents at resonance frequencies while different loads are used; (**a**)—load equal to 1 MΩ; (**b**)—load equal to 500 kΩ; (**c**)—load equal to 250 kΩ.

Figure 20 shows that the maximum output current characteristics were obtained at the third resonance for all resistive load cases. The output current values reached 313.2 μA, 482.18 μA and 876.24 μA for 1 MΩ, 500 kΩ and 250 kΩ, respectively. Compared to the results of numerical investigation, the values have a difference of approximately 21%. However, the characteristics over angular range are more stable compared to the results of numerical investigation. Despite to difference of maximum values, the trends of output current characteristics are similar and shows the energy harvester’s possibility provides output characteristics over the whole angular range. On the other hand, the maximum values do not fully represent the characteristics of the energy harvester. Therefore, in order to illustrate it, the average output current characteristics over the whole angular range of resonance frequencies were calculated. The summary is given in Figure 21.

As can be found in Figure 21, the highest average output current characteristics were obtained at the third resonance frequency. The difference between values at different resonance frequencies are notable but in the same range. On the other hand, the deviations of the average output current values indicates that the stability of the characteristics of the energy harvester over the angular range is mid-level. Despite this, it shows that the energy harvester is able to propose suitable operations over the whole angular range.

**Figure 22:**
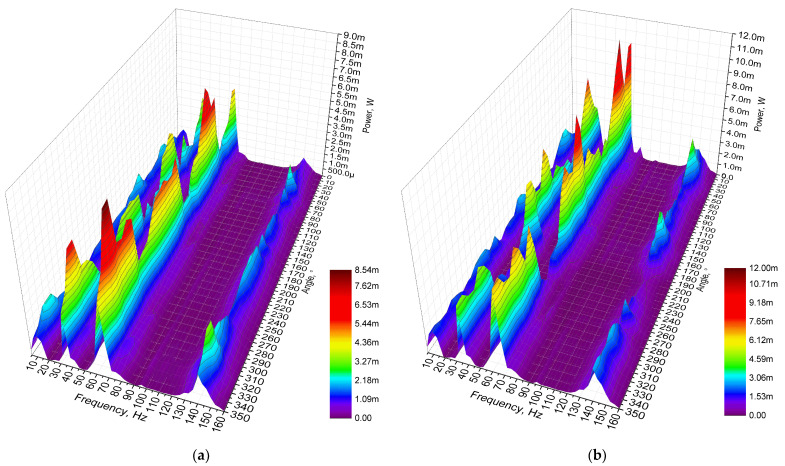

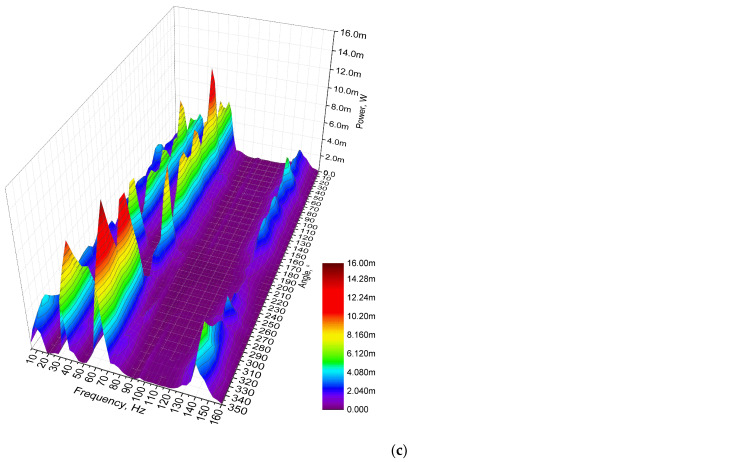
Frequency—power—angle of excitation motion characteristics of the energy harvester with different loads; (**a**)—load equal to 1 MΩ; (**b**)—load equal to 500 kΩ; (**c**)—load equal to 250 kΩ.

The next stage of experimental investigation was performed with the goal to indicate the output power characteristics of the energy harvester over the angular range while different resistive loads were applied. The amplitude of input vibrations, resistive loads and other boundary conditions were set as in previous experimental investigations. The results of the measurements are given in Figure 22.

**Figure 23:**
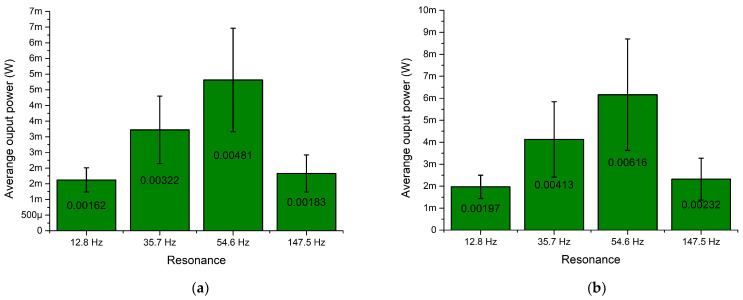

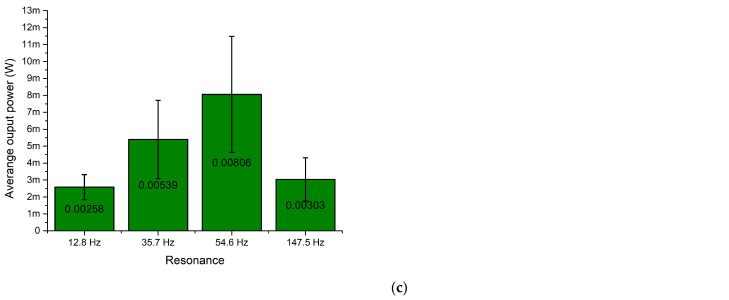
Summary of average output power at resonance frequencies while different loads are used; (**a**)—load equal to 1 MΩ; (**b**)—load equal to 500 kΩ; (**c**)—load equal to 250 kΩ.

Output power characteristics which are presented in Figure 22 show that the highest output power values were obtained at the third resonance. The highest values were obtained at all resistive load values and reached 8.54 mW, 11.6 mW and 15.34 mW at 1 MΩ, 500 kΩ and 250 kΩ, respectively. The lowest maximum output power values were obtained at the first resonance and reached 2.51 mW, 3.39 mW and 4.48 mW at 1 MΩ, 500 kΩ and 250 kΩ, respectively. On the other hand, the maximum values at the fourth resonance frequency are higher around 2–3%. Therefore, at these frequencies, the output power characteristics will be similar. However, the represented output power values are the maximum obtained and do not fully represent the harvester’s operation via the whole angular range. Therefore, a summary of the average output power values at resonance frequencies via the whole angular range was made, and the deviations of the values were calculated in order to fully represent the output power characteristics of the energy harvester. The summary is given in Figure 23.

As shown in the summary of the average output power characteristics at resonance frequencies (Figure 23), the highest values were obtained at the third resonance for all resistive loads. In addition, the average output power values at other resonance frequencies are also significant and are able to provide suitable output characteristics. On the other hand, the deviations of the values over the angular range are notable at all resonances and have influence over the stability of the output power over the angular range. Therefore, considering the results, it can be noted that the energy harvester will be able to provide mid-level stability output characteristics.

In addition, output power characteristics of the energy harvester shows that the introduced design of the energy harvester is able to propose a mid-level stability power supply to the load while the angle of excitation motion changes.

## 5. Conclusions

Considering to the results of numerical and experimental investigations, it can be concluded that the proposed design of the energy harvester is able to provide the bi-directional, multi-modal operation principle via a wide angular range. Moreover, it was found that the proposed design of energy harvester ensures the possibility to obtain relativity stable output characteristics while the frequency and direction of input vibrations have unsystematic and unpredictable changes. It is a result of the composition of the bi-directional and multi-modal operation principle in one structure. In addition, the design of the energy harvester ensures the transfer of stresses which are inducted at resonating cantilevers to cantilevers which are out of resonance mode and thus increases the employment of piezo ceramics which are used to compose the system. In addition, the design of energy harvester consists of a Z-shaped seismic mass which is used to induct additional stresses via the shifted center of the mass. This solution leads to more stable output characteristics via the whole angular range.

Modal analysis showed that the energy harvester has four resonance frequencies at a range from 10 Hz to 180 Hz. The modal shapes of the energy harvester are based on bending modes of the cantilevers, which are used to compose the structure, or compounds of the bending modes. Frequency response analysis confirmed the results of modal analysis and showed that the energy harvester is able to provide output characteristics via the whole angular range with mid-level stability. Experimental investigations verified results of numerical studies and showed that the energy harvester is able to provide electrical characteristics with mid-level stability while the angle of input vibrations changes. Additionally, the experimental investigations showed that the maximum average output power of the energy harvester reaches 8.06 mW with a deviation of 3.42 mW via the whole angular range. In addition, the average energy conversion efficiency at resonance frequencies varies from 0.05 to 0.23. The efficiency of the system is directly related to the angle of input vibrations as well as to the deformation mode. However, the results show that the energy harvester is able to provide suitable output characteristics with acceptable efficiency.

## Figures and Tables

**Table 1:** Geometrical parameters of bi-directional multi-modal energy harvester.

Parameter	Value	Description
W	93 mm	Width of the bi-directional multi-modal energy harvester
H	73 mm	Height of the bi-directional multi-modal energy harvester
L_1_	39.5 mm	Length of the first piezoelectric cantilever
L_2_	79 mm	Length of the second piezoelectric cantilever
L_3_	39.5 mm	Length of the third piezoelectric cantilever
t	1.5 mm	Total thickness of piezoelectric cantilevers
t_b_	0.5 mm	Thickness of cantilevers’ passive layer
t_pzt_	0.5 mm	Thickness of piezoceramic used to compose cantilevers
α_1_	22.5°	Angle of inclination of the first cantilever
α_2_	67.5°	Angle of inclination of the second cantilever
α_3_	67.5°	Angle of inclination of the third cantilever

**Table 2:** Material characteristics.

Material Properties	Beryllium Bronze C17200	PI Ceramics PIC255
Density, (kg/m^3^)	8250	7800
Young’s modulus, (N/m^2^)	1.28 × 10^11^	7.6 × 10^10^
Poisson‘s coefficient	0.33	0.34
Isotropic structural loss factor	0.02	-
Relative permittivity	-	ε_11_^T^/ε_0_ = 1750 ε_33_^T^/ε_0_ = 1800
Elastic compliance coefficient (10^−12^ m^2^/N)	-	S_11_^E^ = 16 S_33_^E^ = 19
Elastic stiffness coefficient c_33_^D^, (N/m^2^)	-	15.4 × 10^10^
Piezoelectric constant d_33_ (10^−12^ m/V)	-	400
Piezoelectric constant d_31_ (10^−12^ m/V)	-	−180
Piezoelectric constant d_15_ (10^−12^ m/V)	-	550

## Data Availability

Not applicable.
